# IL-6–HaloTag^®^ enables live-cell plasma membrane staining, flow cytometry, functional expression, and de-orphaning of recombinant odorant receptors

**DOI:** 10.14440/jbm.2017.206

**Published:** 2017-11-03

**Authors:** Franziska Noe, Tim Frey, Julia Fiedler, Christiane Geithe, Bettina Nowak, Dietmar Krautwurst

**Affiliations:** Deutsche Forschungsanstalt für Lebensmittelchemie – Leibniz Institut, D-85354 Freising, Germany

**Keywords:** cAMP assay, flow cytometry, N-terminal epitope tags, Rho-tag

## Abstract

The assignment of cognate odorant/agonist pairs is a prerequisite for an understanding of odorant coding at the receptor level. However, the identification of new ligands for odorant receptors (ORs) in cell-based assays has been challenging, due to their individual and rather sub-optimal plasma membrane expression, as compared with other G protein-coupled receptors. Accessory proteins, such as the chaperone RTP1S, or Ric8b, have improved the surface expression of at least a portion of ORs. Typically, recombinant ORs carry N-terminal tags, which proved helpful for their functional membrane expression. The most common tag is the ‘Rho-tag’, representing an N-terminal part of rhodopsin, but also ‘Lucy-’ or ‘Flag-tag’ extensions have been described. Here, we used a bi-functional N-terminal tag, called ‘interleukin 6 (IL-6)-HaloTag^®^’, with IL-6 facilitating functional cell surface expression of recombinant ORs, and the HaloTag^®^ protein, serving as a highly specific acceptor for cell-impermeant or cell-permeant, fluorophore-coupled ligands, which enable the quantification of odorant receptor expression by live-cell flow cytometry. Our experiments revealed on average an about four-fold increased surface expression, a four-fold higher signaling amplitude, and a significantly higher potency of odorant-induced cAMP signaling of six different human IL-6-HaloTag^®^-ORs across five different receptor families in NxG 108CC15 cells, as compared to their Rho-tag–HaloTag^®^ constructs. We observed similar results in HEK-293 cells. Moreover, screening an IL-6–HaloTag^®^-odorant receptor library with allyl phenyl acetate, revealed both known receptors as best responders for this compound. In summary, the IL-6–HaloTag^®^ represents a promising tool for the de-orphaning of ORs.

## INTRODUCTION

Odorant receptors (ORs) are seven transmembrane domain G-protein coupled receptors (GPCRs), which are the molecular basis for our sense of smell. Already identified in 1991 [1], the majority (85%) of the ca. 400 different human OR types have no assigned cognate agonists, yet. This is based on a sub-optimal plasma membrane expression of recombinant ORs in heterologous cell systems. ORs may be retained in the endoplasmic reticulum (ER), or are post-translationally modified, and so targeted for degradation [2,3]. Moreover, ORs are prone to be affected by single nucleotide polymorphisms (SNPs), which may alter ligand binding, signaling, or trafficking to the plasma membrane [4-6]. Beyond measuring their functional expression, it is therefore inevitable to investigate also the plasma membrane expression of ORs and their genetic variants. Research on intracellular OR trafficking focused primarily on the role of forward trafficking proteins [3]. These include accessory proteins such as receptor-transporting protein 1 (RTP1), or RTP1S, RTP2, receptor expression-enhancing protein 1 (REEP1) [7,8], and the guanine nucleotide exchange factor Ric8b [9], which enhanced the cell surface expression of recombinant ORs in test cell systems without altering their ligand specificity [10]. Still, an enhanced cell surface expression often requires a modification of the OR itself: it is, therefore, common to fuse the receptor’s N-terminus with a protein ‘tag’. Such epitope tags are short peptide sequences with useful characteristics. First, high-affinity antibodies and/or chemical labels [11] are usually available for tags, which enable the detection and visualization of recombinant protein in experimental setups such as western blotting, immunocytochemistry, or immunoprecipitation. Common tags are, for example, FLAG-tag [12] or His-tag [13]. Second, N- or C-terminal tags may facilitate the cell-surface expression of recombinant membrane proteins in heterologous cell systems. For ORs, several tags have been used to improve their sub-optimal plasma membrane expression. Initially, the addition of the first 20 amino acids of bovine rhodopsin to the N-terminus of ORs, called Rho-tag, were shown to enable the surface expression of ORs in test cell systems [14]. This Rho-tag, or an extended version, comprising the entire N-terminus of rhodopsin, has been validated to facilitate cell surface expression of recombinant ORs by several groups independently, and for a variety of chemosensory odorant and taste receptors [10,15-18], and today is the most commonly used N-terminal tag for recombinant ORs, but also for taste receptors. Shepard *et al*. [19] investigated a leucine-rich 17-amino acid cleavable signal peptide (LRRC32), which they named “Lucy”. Further, they combined Lucy-tag with Rho-tag and could show, that the combination of Lucy-tag with Rho-tag increased the surface expression of all investigated ORs in their study [19].

In this study, we used a bi-functional N-terminal tag, that consists of two parts, interleukin 6 (IL-6) and a modified bacterial haloalkane dehalogenase, the HaloTag^®^, which can be recognized by several cell-impermeant or cell-permeant fluorophore-coupled ligands [20,21]. The HaloTag^®^ technology comprises the HaloTag^®^ protein and a variety of organic molecules, the HaloTag^®^ ligands, which irreversibly bind to the HaloTag^®^ protein. Under physiological conditions, this covalent bond forms rapidly and is highly specific. The HaloTag^®^ protein is a 33 kDa monomeric protein that does not occur naturally in mammals, plants, or *E. coli*. Therefore, its nonspecific activity is extremely low [20,21]. IL-6 is a secreted protein (~25 kDa) of the immune system. It belongs to the group of pro-inflammatory cytokines and plays a key role in the unspecific, innate immune response. Synthesis and release of IL-6 are initiated by the binding of foreign antigens to the receptors of the innate immunity, and appear within few hours after the activation of the cellular immune system [22].

Here we compared several IL-6-HaloTag^®^-OR constructs with known agonists with their Rho-tag-HaloTag^®^ constructs with respect to their functional plasma membrane expression in NxG 108CC15 and HEK-293 cells. To evaluate their screening performance and concentration-response relations we used the fast, online luminescence-based GloSensor^®^ cAMP assay [15,23]. We assessed the plasma membrane expression of all OR constructs by live-cell staining with a cell-impermeable, fluorophore-coupled HaloTag^®^ ligand, and subsequent analysis by flow cytometry.

## METHODS

### Chemicals

The following chemicals were used: Dulbecco’s MEM medium (#F0435), FBS superior (#S0615), L-glutamine (#K0282), penicillin (100U/ml)/streptomycin (100U/ml) (#A2212), trypsin/EDTA solution (#L2143) (Biochrom, Berlin, Germany), MEM non-essential amino acid solution (100x) (#M7145, Sigma-Aldrich, Steinheim, Germany), Gibco^®^ HAT supplement (#21060-017, Thermo Fisher, Dreieich, Germany),CaCl_2_*2H_2_O (#22322.295), D-glucose (#101174Y), dimethyl sulfoxide (DMSO) (#83673.230), HEPES (#441476L), potassium chloride (#26764.230), and sodium hydroxide (#28244.295) (VWR Chemicals BDH Prolabo, Leuven, Belgium), sodium chloride (#1064041000, Merck, Darmstadt, Germany), D-luciferin (beetle) monosodium salt (#E464X), HaloTag^®^ Alexa Fluor^®^ 488 Ligand (#G1001, Promega, Madison, USA), Dynasore (#2897, Tocris Bioscience, Bristol, UK), Hoechst33342 (#1399, Invitrogen, Eugene, USA), Mowiol 4-88 (#0713, Carl Roth GmbH + Co. KG, Karlsruhe, Germany), Paraformaldehyde (PFA) (#18814, Polysciences Inc., Warrington, USA).

Odorants were purchased from Sigma-Aldrich (Steinheim, Germany), Alfa-Aesar (Karlsruhe, Germany) and Chemos GmbH (Regenstauf, Germany) (**Table S1**).

### Molecular cloning

The protein-coding regions (NCBI reference sequence, **Table S2**) of human OR1A1, OR2M3, OR2W1, OR8D1, OR10J5, OR51E1 and mouse Olfr16 were amplified from genomic DNA with gene specific primers (**Table S3**) by polymerase chain reaction (PCR).

PCR reactions with a final volume of 50 μl were performed in a C-1000 thermocycler (BioRad, Muenchen, Germany) with 150 ng of respective genomic DNA, 0.5 μl Phusion hot start DNA-polymerase (#F-534L), 1.5 μl DMSO (#F-515, Thermo Scientific, Waltham, USA), 2.5 mmol/L dNTPs (#U1511, Promega, Madison, USA), and 0.5 μmol/L of each primer. The following protocol was used: denaturation (98°C, 3 min), followed by 10 cycles containing: denaturation (98°C, 30 s), annealing with 1°C decreasing in temperature each cycle (start 66°C, 30 s), extension (72°C, 2 min); 30 cycles containing: denaturation (98°C, 30 s), annealing (58°C, 30 s), extension (72°C, 2 min) and final elongation (72°C, 10 min).

PCR products were purified (gel extraction kit, #28706, Qiagen, Hilden, Germany), digested either EcoRI/NotI (#R6017/ #R6435, Promega, Madison, USA) or MfeI/NotI (#R0589S/ #R0189S, New England Biolabs, Ipswich, USA) and ligated with T4 DNA ligase (#M1804, Promega, Madison, USA) into the expression plasmid pFN210A (#pFN210A SS-HaloTag^®^ CMV-neo Flexi^®^-Vector, Promega, Madison, USA).

Plasmid-DNA was transformed by heat shock in competent *E. coli* XL-1-blue cells (#200249, Agilent Technologies, Santa Clara, USA) and purified with pure yield plasmid midiprep kit (#A2495, Promega, Madison, USA). Plasmid-DNA concentration was determined with the Nanodrop 2000 (Thermo Fisher Scientific, Waltham, USA) and adjusted to 250 ng/μl.

### Sequencing

All sub-cloned wild-type (wt.) and mutated OR-coding amplicons were verified by Sanger sequencing (Eurofins Genomics, Ebersberg, Germany) by using vector internal primers (**Table S4**).

### Cell culture and transient DNA transfection

We used a neuroblastoma x glioma hybrid (NxG 108CC15 cells), and a human embryonic kidney (HEK-293 cells) cell line, as test cell systems for the functional expression of recombinant ORs as described previously [15]. The transfection was performed by using the lipofection method with either the IL-6-HaloTag^®^-OR or the Rho-HaloTag^®^-OR constructs. The transport protein RTP1S [7], G protein subunit Gαolf [24,25], olfactory G protein subunit Gγ13 [26] and the GloSensor^TM^-22F [23] (Promega, Madison, USA) were co-transfected using Lipofectamine^®^ 2000 (#11668-027, Life Technologies, USA). The pGloSensor^TM^-22F is a genetically engineered luciferase with a cAMP binding pocket, which allows measuring a direct cAMP dependent luminescence signal. As a negative control, we transfected the vector plasmid pFN210A lacking any OR coding region, together with Gαolf, RTP1S, Gγ13 and cAMP-luciferase pGloSensor^TM^-22F (mock). The amount of transfected plasmid-DNA was equal in OR-transfected and mock-transfected cells.

### cAMP luminescence assay

The cAMP luminescence assays were performed 42 h post transfection as reported previously [15]. The cells were incubated with a physiological salt buffer (pH 7.5) containing 140 mmol/L NaCl, 10 mmol/L HEPES, 5 mmol/L KCl, 1 mmol/L CaCl_2_, 10 mmol/L D-glucose and 2% D-luciferin. For the cAMP luminescence measurements, the Glomax^®^ MULTI^+^ detection system (Promega, Madison, USA) was used. After an incubation of the cells for 1 h in the dark, the basal luminescence signal of each well was recorded. Odorant stock solutions were prepared in DMSO and diluted 1:1000 in the physiological salt buffer to obtain a final DMSO concentration of 0.1% DMSO on the cells. Real-time luminescence signals for each well were measured 4 min after the odorant application.

### Data analysis of cAMP luminescence measurements

The raw luminescence data obtained from the Glomax^®^ MULTI^+^ detection system were analyzed using Instinct Software (Promega, USA). Three data points before (baseline) and after odorant addition (signal) were averaged, and the respective baseline value was subtracted from each signal. For screening experiments, a single experiment was performed and two data points were averaged. The signals were normalized by the highest luciferase ratio and a 3σ-threshold was calculated. All signals above the 3σ-threshold (mean + 3 standard deviations over all signals) were assessed as positive signals. For concentration-response relations, the baseline-corrected dataset was normalized to the maximum amplitude of each odorant-receptor pair. EC_50_ values and curves were derived from fitting the function f(*x*) = ((min–max)/(1 + (*x*/EC_50_)^hillslope^)) + max to the data by nonlinear regression (SigmaPlot 10.0, Systat Software). All data are presented as mean ± SD.

### Flow cytometry

NxG 108CC15 cells [27] were cultivated in 12-well plates (80000 cells/well) and transiently transfected with 800 ng plasmid-DNA of the respective OR as well as 400 ng plasmid-DNA of Gαolf, Gγ13, RTP1S and cAMP-luciferase pGloSensor^TM^-22F each using Lipofectamine^®^ 2000. To keep the amount of transfected DNA compared to cell number the same as in the luminescence assay, we also transfected the cAMP-luciferase pGloSensor^TM^-22F, although it has no impact on cell surface expression of the respective OR. Basically, the experimental settings were scaled up 8-fold from the 96-well luminescence assay to the 12-well flow cytometry assay. As a control, the transfection was performed with the vector plasmid pFN210K which is lacking the coding information of the HaloTag^®^ (mock control).

For analysis, cells were harvested 42 h post transfection and stained with the cell-impermeant HaloTag^®^ Alexa Fluor^®^ 488 Ligand (ex/em = 499/518 nm). Cells were incubated for 1 h at 37°C and 7% CO_2_ in the cell culture incubator. Cells were washed twice with serum free medium prior to FACS analyses (MACSQuant Analyzer, Miltenyi Biotec, Bergisch Gladbach, Germany). A forward- and side-scatter gate was set to exclude dead cells with forward-scatter (FSC: 235V) and side-scatter (SSC: 360V). The FITC signal (B1-channel; HaloTag^®^ Alexa Fluor488 Ligand) was detected with 175V. In each case 10000 cells were measured. The analysis was performed with the MACSQuantify software (Miltenyi Biotec, Bergisch Gladbach, Germany). The FITC signal of each mock control (AlexaFluor488 treated cells) defined the distinction between negative and positive cells.

### Membrane-impermeable fluorescence labeling of cell surface HaloTag^**^®^**^ **ORs**

NxG 108CC15 cells [27] were cultivated in 6-well plates (300000 cells/well) and transiently transfected with 3000 ng plasmid-DNA of the respective OR as well as 1500 ng plasmid-DNA of Gαolf, Gγ13, RTP1S and cAMP-luciferase pGloSensorTM-22F each using Lipofectamine^®^ 2000. To keep the amount of transfected DNA compared to cell number the same as in the luminescence assay, we also transfected the cAMP-luciferase pGloSensor^TM^-22F, although it has no impact on cell surface expression of the respective OR. As a control, the transfection was performed with the vector plasmid pFN210K which is lacking the coding information of the HaloTag^®^ (mock control).

For analysis, 40 h post transfection cells were incubated with or without 80 μM Dynasore in DMSO for 30 min at 37°C and 7% CO_2_. Throughout the following steps the concentrations of Dynasore and/or DMSO were kept constant. Cells were washed once and harvested. After centrifugation the cells were incubated with the cell-impermeant HaloTag^®^ Alexa Fluor^®^ 488 Ligand (ex/em = 499/518 nm) for 1 h at 37°C and 7% CO_2_. Cells were washed once with serum free medium and stained with Hoechst33342 (1:1000) for 7 min in the dark. After centrifugation the cells were fixed with 2% PFA for 15 min at RT. The cells were washed again, divided in half, and centrifuged. For FACS analyses, cells were re-suspended in serum free medium, and for confocal microscopy, cells were re-suspended in Mowiol 4-88, and transferred to a cover slip.

Fluorescence confocal microscopy was performed on a laser-equipped, inverted Olympus FV1000 microscope with an Olympus UPlanSApo 60x/w objective.

## RESULTS

In 2015, we introduced the fast, online GloSensor^TM^ [23] technology as a useful tool for the sensitive detection of odorant/receptor-induced cAMP signaling in recombinant test cell systems, such as HEK-293 or NxG 108CC15 cells [15]. Since then, we and others have successfully used the GloSensor^TM^ cAMP assay for the de-orphaning and functional characterization of ORs in recombinant test cell systems [6,15,17,28]. In the present study, we used NxG 108CC15 cells, which actually express RNA for olfactory adenylyl cyclase (AC) type III, as well as for four other ACs (**Table S5**, **Fig. S1**, [15]), together with the GloSensor^TM^ assay, to measure the function of seven ORs and one non-olfactory GPCR, carrying different N-terminal tags.

### Different N-terminal epitope tags have a profound influence on the ligand specificity of ORs

It was commonly observed that the use of the Rho-tag [14] as N-terminal epitope tag for ORs enhances their cell-surface expression [14,18]. Here we used another tag, called HaloTag^®^ combined with IL-6 (IL-6-HaloTag^®^) and compared the function of well-known, de-orphaned ORs carrying this tag with ORs carrying the HaloTag^®^ combined with Rho-tag (Rho-tag-HaloTag^®^), but also with ORs carrying just the Rho-tag.

Therefore we established concentration-response-relations for six ORs of different families, one OR of mice as well as dopamine receptor D_1_ (DRD1).

**Figure 1.**
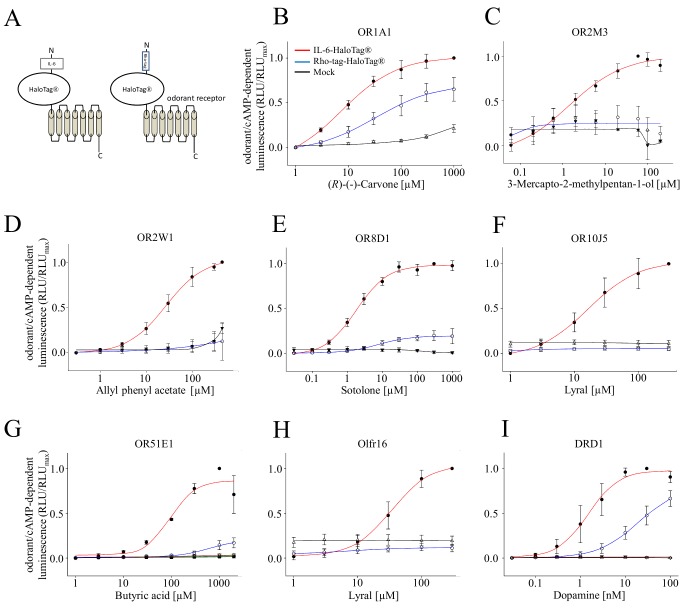
Concentration-response relations for selected odorant/OR combinations with different N-terminal tags in NxG 108CC15 cells. **A.** Schemes of receptor constructs, carrying either IL-6-HaloTag^®^ or Rho-tag-HaloTag^®^. **B.** OR1A1 with (R)-(-)-carvone. **C.** OR2M3 with 3-mercapto-2-methylpentan-1-ol. **D.** OR2W1 with allyl phenyl acetate. **E.** OR8D1 with sotolone. **F.** OR10J5 with lyral. **G.** OR51E1 with butyric acid. **H.** Olfr16 with lyral [29]. **I.** DRD1 with dopamine. EC_50_ values (**Table 1**) were derived from fitting a function to concentration-response data by means of non-linear regression. Shown are mean ± SD (*n* = 3–5). Data were normalized to each receptor’s maximum amplitude with the N-terminal tag IL-6-HaloTag^®^. Red colored curves indicate receptors with the N-terminal tag IL-6-HaloTag^®^, blue with Rho-tag-HaloTag^®^, and black curves indicate the empty plasmid control (Mock).

We could observe for all tested receptors, which were tagged with IL-6-HaloTag^®^, lower EC_50_ values as well as higher amplitudes, as compared to their corresponding Rho-tag-HaloTag^®^ constructs (**Fig. 1**, **Table 1**). Moreover, ORs carrying the IL-6-HaloTag^®^ in general outperformed ORs carrying just the Rho-tag (**Fig. S2**), with the exception of OR2M3, where the Rho-tagged receptor outperformed all other tags (**Fig. S2C**).

**Figure 2.**
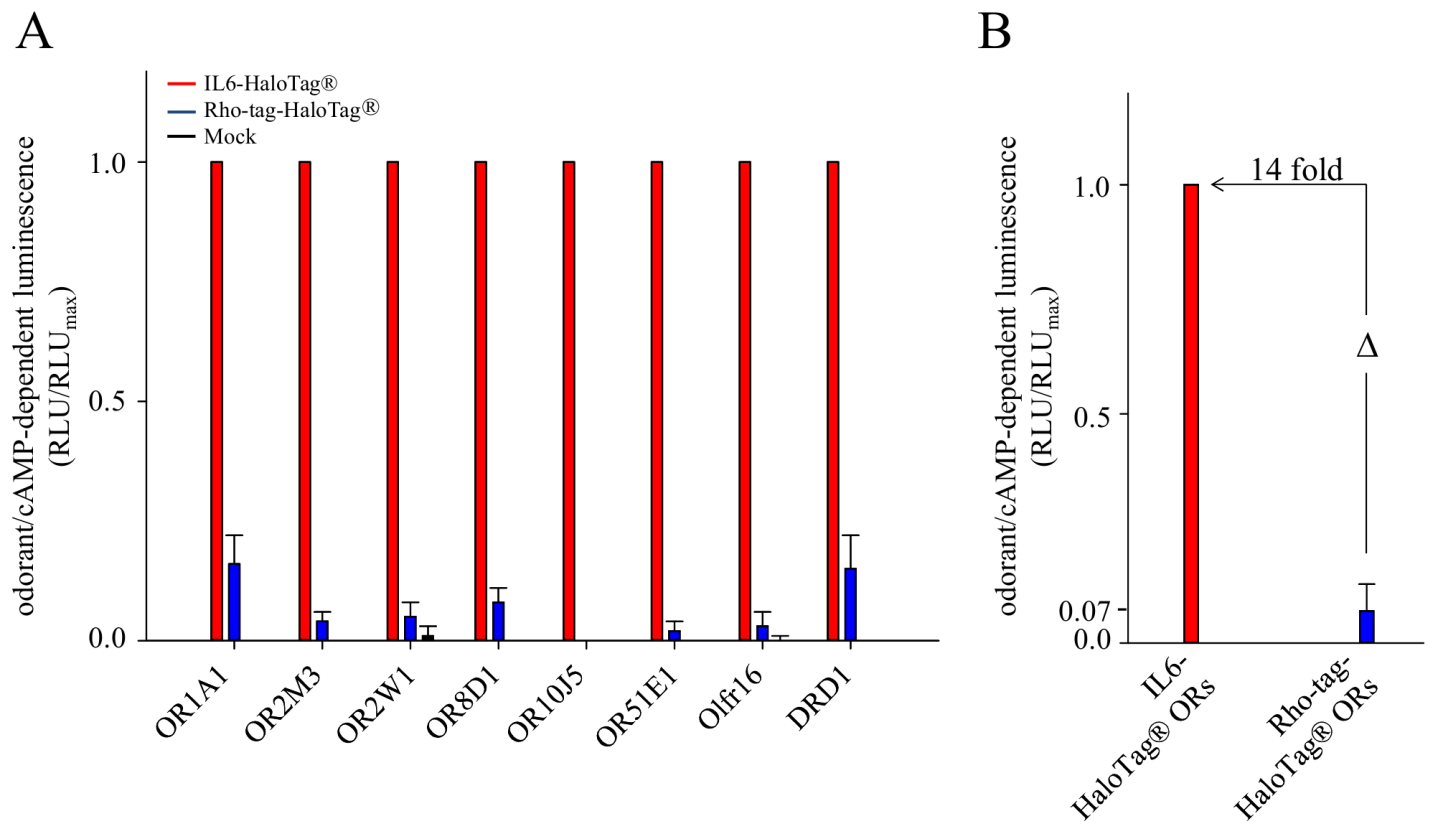
Normalized efficacies of receptors carrying the epitope tags IL-6-HaloTag^®^ or Rho-tag-HaloTag^®^ in HEK-293 cells. **A.** Shown are the normalized amplitudes of receptors carrying either the IL-6-HaloTag^®^ (red bars) or the Rho-tag-HaloTag^®^ (blue bars), when challenged with their reported agonist: OR1A1 with (R)-(-)-carvone (1000 μM); OR2M3 with 3-mercapto-2-methylpentan-1-ol (60 μM); OR2W1 with allyl phenyl acetate (300 μM); OR8D1 with sotolone (300 μM); OR10J5 with lyral (300 μM); OR51E1 with butyric acid (300 μM); Olfr16 with lyral (300 μM); and DRD1 with dopamine (10 nM). Shown are means ± SD (*n* = 3). Data were normalized to each receptor’s maximum amplitude with the N-terminal tag IL-6-HaloTag^®^. Mock, empty plasmid control (black bars). **B.** Shown are differences in odorant responses averaged over all tested ORs carrying either the IL-6-HaloTag^®^ (red bar) or the Rho-tag-HaloTag^®^ (blue bar).

Further, we compared the above investigated receptors also in HEK-293 cells. Here we screened the receptors, which were equipped with different epitope tags, with the same, single concentration of their reported agonist, and compared their normalized response amplitudes. We observed higher amplitudes across the investigated receptors carrying the new epitope tag IL-6-HaloTag^®^, as compared to their Rho-tag-HaloTag^®^ constructs (**Fig. 2**).

HaloTag^®^ technology enables the live-cell cell-surface staining and flow cytometry, and intracellular monitoring of labeled receptors

**Figure 3.**
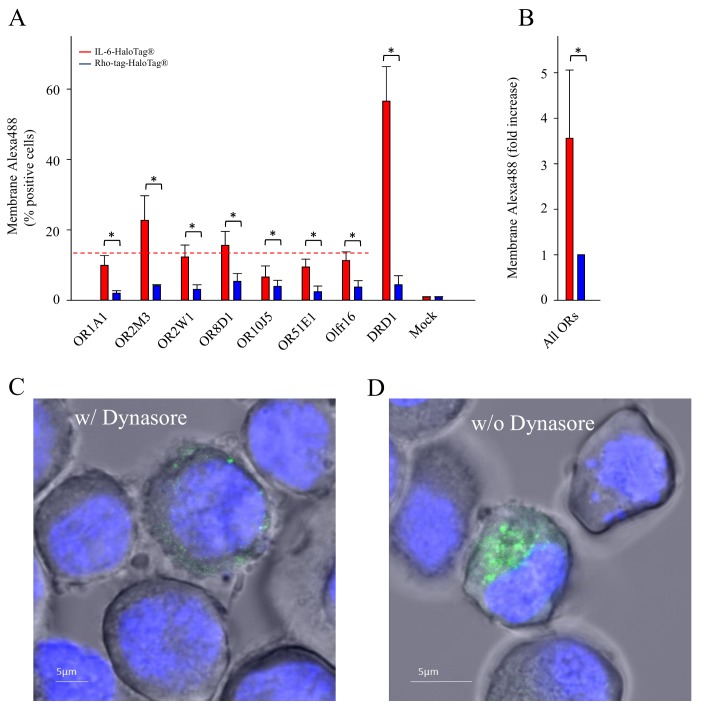
Analysis of the cell surface expression of investigated ORs with both N-terminal tags. **A.** Flow cytometry analysis of transiently transfected NxG 108CC15 cells showing a detectable fluorescence of membrane-impermeable HaloTag^®^ Ligand-Alexa488, suggesting different cell surface expression of ORs carrying different N-terminal tags. Data are means ± SD (*n* = 3–6). Mock, empty plasmid control. *P < 0.05 as compared to Rho-tag-HaloTag^®^. **B.** Differences averaged over all tested ORs, carrying either the IL-6-HaloTag^®^ (red bar), or the Rho-tag-HaloTag^®^ (blue bar), and normalized to the Rho-tag-HaloTag^®^ ORs. *P < 0.05 as compared to Rho-tag-HaloTag^®^. **C.** Fluorescence confocal image of NxG 108CC15 cells expressing IL-6-HaloTag^®^-OR8D1, labeled with membrane-impermeable HaloTag^®^ Ligand-Alexa488, and treated with endocytosis blocker Dynasore. **D.** Fluorescence confocal image of NxG 108CC15 cells expressing IL-6-HaloTag^®^-OR8D1, labeled with membrane-impermeable HaloTag^®^ Ligand-Alexa488, but without endocytosis blocker Dynasore.

It has been repeatedly observed that different OR types do not express equally well at the plasma membrane in recombinant test cell systems. For receptors carrying the HaloTag^®^, however, we were able to measure the cell-surface expression of ORs *via* flow cytometry. We investigated six human ORs, one mouse OR and DRD1 by labeling the HaloTag^®^ with a cell-impermeant ligand (HaloTag^®^ Alexa Fluor^®^ 488 Ligand). We observed, that all tested receptors carrying the IL-6-HaloTag^®^, showed a higher cell-surface expression than their Rho-tag-HaloTag^®^ constructs (**Fig. 3**, **Table 1**).

We observed cell surface expression across all tested ORs on average in about 13% of NxG 108CC15 cells (red dashed line in **Fig. 3A**). In contrast, we observed cell surface expression of DRD1 in about 57% of cells (**Fig. 3A**). The transfection efficiency across all ORs consistently was 30% in NxG 108CC15 cells (data not shown). Blocking a clathrin-dependent endocytosis by Dynasore, we observed membrane-impermeable HaloTag^®^ Ligand-Alexa488-dependent fluorescence, typically accumulated in cell surface clusters (**Fig. 3C**). In contrast, under control conditions, where a clathrin-dependent endocytosis was not blocked, a membrane-impermeable HaloTag^®^ Ligand-Alexa488-dependent fluorescence typically accumulated within cells, presumably in endosomes (**Fig. 3D**). Flow cytometry revealed that cells from these two experimental conditions segregated with respect to their fluorescence intensity distributions, with endocytosis-blocked cells shifting to higher fluorescence intensities (**Fig. S3E**, **Table S6**).

### Accessory proteins influence the cell-surface expression of ORs

**Figure 4.**
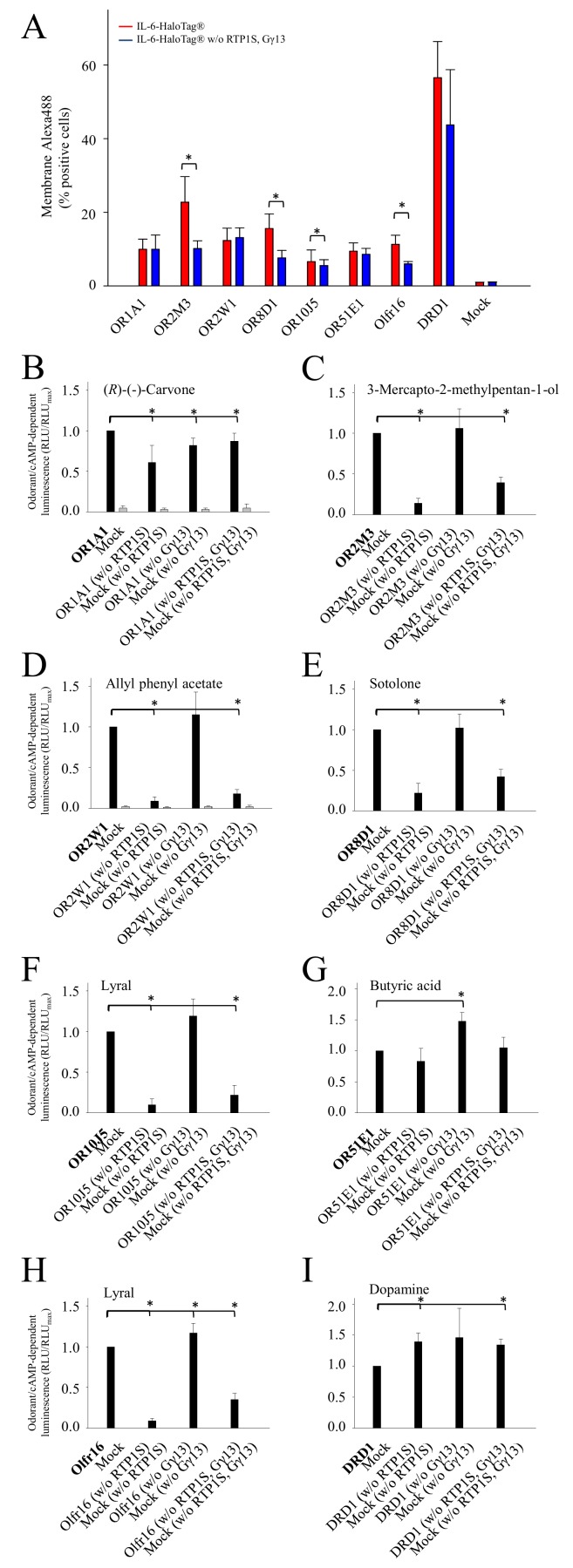
The influence of accessory proteins on the OR cAMP signaling in NxG 108CC15 cells. Flow cytometry analysis of the cell surface expression of investigated ORs (A). The data represent the percentage of NxG 108CC15 transiently transfected cells with a detectable membrane-impermeable HaloTag^®^ Ligand-Alexa488-dependent fluorescence, suggesting cell surface expression of OR, as determined by flow cytometry. Red bars indicate the transfection with all accessory proteins and blue bars indicate the transfection without (w/o) RTP1S and Gγ13. Shown are mean ± SD (*n* = 3–6). Mock, empty plasmid control. cAMP-luminescence measurements of OR1A1 with (R)-(-)-carvone (1000 μM) (B), OR2M3 with 3-mercapto-2-methylpentan-1-ol (60 μM) (C), OR2W1 with allyl phenyl acetate (300 μM) (D), OR8D1 with sotolone (300 μM) (E), OR10J5 with lyral (300 μM) (F), OR51E1 with butyric acid (300 μM) (G), Olfr16 with lyral (300 μM) (H), and DRD1 with dopamine (10 nM) (I). All receptors were tagged with IL-6-HaloTag^®^. Data were normalized to each receptor with co-transfection of RTP1S and Gγ13. Shown are mean ± SD (*n* = 3). Black bars are receptor responses in the presence of RTP1S and Gγ13, and grey bars indicate empty plasmid control (Mock). *P < 0.05 as compared to receptor responses in the presence of RTP1S and Gγ13.

Earlier studies have shown that RTP1S, Ric8b and Gαolf induce the highest functional expression of ORs when combined with Rho-tag [10]. Further, Gγ13 was shown to be important for the functional formation of the heterotrimeric olfactory G-protein [26]. Therefore, we investigated if IL-6-HaloTag^®^ could also work synergistically with the accessory proteins RTP1S and Gγ13. For our tests, we used the IL-6-HaloTag^®^ tagged ORs and co-transfected them with Gαolf. RTP1S and Gγ13 were transfected either individually or co-transfected for luminescence measurements. To investigate the cell surface expression, RTP1S and Gγ13 were co-transfected. Both, the cell-surface as well as the cAMP signaling-dependent luminescence measurements revealed that the accessory proteins RTP1S and Gγ13 differentially and receptor-dependently influenced receptor responses and cell surface expression. The presence of RTP1S and Gγ13 resulted in a significantly higher cell surface expression for OR2M3, OR8D1, OR10J5, and Olfr16, with no effect on OR1A1, OR2W1, OR51E1, or DRD1 (**Fig. 4A**).

OR1A1, activated by (R)-(-)-carvone, showed only a slight but significant decrease in receptor response, if either RTP1S or Gγ13, or both were absent (**Fig. 4B**). In contrast, the OR2M3 response to 3-mercapto-2-methylpentan-1-ol was almost non-detectable without RTP1S, whereas the absence of Gγ13 had no impact (**Fig. 4C**). Similar effects were observed for OR2W1 with allyl phenyl acetate (**Fig. 4D**), for OR8D1 with sotolone (**Fig. 4E**), for OR10J5 with lyral (**Fig. 4F**), as well as for mouse Olfr16 with lyral [29] (**Fig. 4H**). The absence of RTP1S had no impact on the butyric acid function of OR51E1 (**Fig. 4G**), or the dopamine function of DRD1 (**Fig. 4I**), whereas the absence of Gγ13 even appeared to enhance the response amplitude of both receptors.

### OR1A1 and OR2W1 respond best to allyl phenyl acetate among human ORs

**Figure 5.**
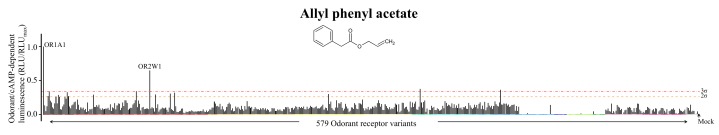
Allyl phenyl acetate at 100 μM activates two receptors out of 579 odorant receptor variants. Screening of allyl phenyl acetate (100 μM) against 579 OR variants in NxG 108CC15 cells. Data (*n* = 1 in duplicates) were normalized to the maximum responding OR (OR1A1). OR families are color-coded and sorted in ascending numerical order. Dashed lines indicate 2- and 3σ-thresholds. Mock, empty plasmid control.

To identify cognate human odorant receptors for the odorant allyl phenyl acetate, we screened this compound at 100 μM against 579 human OR variants carrying the N-terminal IL-6-HaloTag^®^. Notably, allyl phenyl acetate best activated OR1A1 and OR2W1 (**Fig. 5**), which both have been shown to respond to this compound [6,30].

## DISCUSSION

The testing of ORs in recombinant cell systems so far has been challenged by the ORs’ poor expression at the cell surface and sub-optimal signaling, as compared to non-olfactory GPCRs. Moreover, some ORs are more efficiently trafficked to the cell surface than others [19], which we observed, too, in our present study. Here, we report that the introduction of the bi-functional N-terminal IL-6-HaloTag^®^ outperforms the Rho-tag-HaloTag^®^ in terms of cell surface expression of all investigated ORs, and also outperforms commonly used Rho-tag in terms of signaling of six out of seven investigated ORs, in both HEK-293 cells, as well as in the neuroblastoma x glioma NxG 108CC15 cell line. Moreover, both the cell surface expression as well as the potency of dopamine-induced signaling of non-olfactory receptor DRD1 improved significantly in the presence of the N-terminal IL-6-HaloTag^®^, as compared to the Rho-tag-HaloTag^®^. However, with DRD1 and OR2M3, the N-terminal Rho-tag alone outperformed all other tested tags in the GloSensor^TM^ cAMP assay (**Fig. S2C**, **I**). Here, the bulky HaloTag^®^ (33 kDa) may restrain the interaction of the agonist with its receptor.

We did not show membrane staining for the Rho-tag constructs since this would have required antibody staining of paraformaldehyde-fixed cells, which may partially permeabilize cells, and thus would not have been comparable with the live-cell plasma membrane staining of IL-6-HaloTag^®^-ORs. However, just by exchanging the Rho-tag by IL-6 in the Halo-tagged ORs, our flow cytometry experiments suggest IL-6 as the by far more powerful determinant for a cell surface expression of ORs.

Under non-stimulated conditions, IL-6-HaloTag^®^-ORs labeled with membrane-impermeable HaloTag^®^ Ligand-Alexa488 accumulated within cells, suggesting homeostatic endocytosis of cell surface-labeled receptors into endosomes, which was prevented in the presence of Dynasore, a blocker of clathrin-dependent endocytosis. Here, we observed a clustered cell surface staining, suggesting a plasma membrane accumulation of IL-6-HaloTag^®^-ORs labeled with membrane-impermeable HaloTag^®^ Ligand-Alexa488. Our experiments show that IL-6-HaloTag^®^ may be suitable to track the endocytosis of cell surface-labeled ORs, to investigate, for instance, endocytosis of odorant-stimulated ORs.

As reported previously, RTP1S has a chaperone function to promote the surface expression of some ORs, even in the absence of Ric-8b and Gαolf [8,10]. In our hands, RTP1S had a significant impact on the plasma membrane expression of only 4 out of 7 tested ORs carrying the N-terminal IL-6-HaloTag^®^. In contrast, 6 out of 7 tested ORs carrying the N-terminal IL-6-HaloTag^®^ showed a significantly reduced signaling in the absence of RTP1S, using the GloSensor^TM^ cAMP assay. This suggests that an increased cell surface expression of ORs by the N-terminal IL-6-HaloTag^®^ supersedes the chaperone function of RTP1S, at least for some ORs, and/or the function of RTP1S is rather to facilitate cAMP signaling than chaperoning of ORs. Further studies are required to understand the plasma membrane trafficking of ORs.

In summary, our study shows that the use of the N-terminal IL-6-HaloTag^®^ on ORs largely improves their functional expression in different test cell systems, using flow cytometry and the fast, online GloSensor^TM^ cAMP assay. Both methods are described in detail by Noe *et al* [31]. This experimental strategy may facilitate the de-orphaning of ORs, and thus will lead to an understanding of odorant coding at the receptor level.

## Supplementary Material

Supplementary information **Figure S1.** RT-PCR of adenylyl cyclases in NxG cells.
**Figure S2.** Concentration-response relations for selected odorant /OR combinations with three different N-terminal tags in NxG 108CC15 cells.
**Figure S3.** Flow cytometry analysis of 10,000 NxG 108CC15 cells expressing IL-6-HaloTag^®^-OR8D1 and labelled with cell membrane impermeant HaloTag^®^ Ligand-Alexa488.
**Table S1**. Investigated compounds.
**Table S2**. NCBI reference sequences of investigated odorant receptor genes.
**Table S3**. Oligonucleotides for molecular cloning of investigated receptors.
**Table S4**. Vector internal oligonucleotides for pFN210A.
**Table S5**. Oligonucleotides for RT-PCR of adenylyl cyclases in NxG cells.
**Table S6**. Numbers from **Fig. S3E**.
Supplementary information of this article can be found online at
http://www.jbmethods.org/jbm/rt/suppFiles/206.

## Figures and Tables

**Table 1.** EC_**50**_ values, efficacies and membrane staining of different receptors in pFN210A and pFN210A-Rho-tag.

Receptor^a^	IL-6–HaloTag^®^–receptor			Rho-tag–HaloTag^®^–receptor		
	EC_50_ [μM]	Efficacy^b^	Cell surface expression^c^	EC_50_ [μM]	Efficacy^b^	Cell surface expression^c^
OR1A1	7.66 ± 4.75	1.00	9.93 ± 2.76	30.61 ± 4.29	0.65 ± 0.13	2.00 ± 0.73
OR2M3	1.08 ± 1.16	1.00	22.73 ± 6.95	n.d.	0.30 ± 0.14	4.13 ± 0.31
OR2W1	26.95 ± 1.37	1.00	12.30 ± 3.36	n.d.	0.13 ± 0.21	3.08 ± 1.31
OR8D1	1.81 ± 0.19	1.00	15.58 ± 3.96	7.74 ± 0.90	0.20 ± 0.06	5.37 ± 2.21
OR10J5	16.32 ± 1.05	1.00	6.60 ± 3.14	n.d.	0.05 ± 0.02	3.93 ± 1.74
OR51E1	95.59 ± 33.39	1.00	9.44 ± 2.23	< 1000	0.14 ± 0.05	2.41 ± 1.62
Olfr16	33.99 ± 2.21	1.00	11.28 ± 2.47	7.07 ± 6.26	0.11 ± 0.04	3.73 ± 1.84
DRD1^d^	1.51 ± 0.28	1.00	56.57 ± 9.84	20.00 ± 1.49	0.48 ± 0.11	4.40 ± 2.57

^a^Official gene symbols.

^b^normalized to each receptor maximum with N-terminal tag IL-6-HaloTag^®^.

^c^Cell surface expression is given as % of cells with a mock-corrected, cell surface-bound Alexa488 signal of a total of 10000 analyzed cells per receptor.

^d^EC_50_ values in [nM]. Data are given as mean ± SD (*n* = 3–8). n.d. means no response detected up to 1000 μM.
